# The three-year evolution of Germany’s Digital Therapeutics reimbursement program and its path forward

**DOI:** 10.1038/s41746-024-01137-1

**Published:** 2024-05-24

**Authors:** Linea Schmidt, Marc Pawlitzki, Bernhard Y. Renard, Sven G. Meuth, Lars Masanneck

**Affiliations:** 1 Hasso Plattner Institute, Digital Engineering Faculty, University of Potsdam, Potsdam, Germany; 2 Hasso Plattner Institute for Digital Health at Mount Sinai, Icahn School of Medicine at Mount Sinai, New York, NY, USA; 3 Windreich Dept. of Artificial Intelligence & Human Health, Icahn School of Medicine at Mount Sinai, New York, NY, USA; 4 Department of Neurology, Medical Faculty and University Hospital Düsseldorf, Heinrich Heine University Düsseldorf, Düsseldorf, Germany; 5 German Society of Digital Medicine e.V. (DGDM), Berlin, Germany

**Keywords:** Health policy, Therapeutics, Health care economics, Clinical trial design

## Abstract

The 2019 German Digital Healthcare Act introduced the Digital Health Application program, known in German as ‘Digitale Gesundheitsanwendungen’ (DiGA). The program has established a pioneering model for integrating Digital Therapeutics (DTx) into a healthcare system with scalable and effective reimbursement strategies. To date, the continuous upward trend enabled by this framework has resulted in more than 374,000 DiGA prescriptions, increasingly cementing its role in the German healthcare system. This perspective provides a synthesis of the DiGA program’s evolution since its inception three years ago, highlighting trends regarding prescriptions and pricing as well as criticisms and identified shortcomings. It further discusses forthcoming legislative amendments, including the anticipated integration of higher-risk medical devices, which have the potential to significantly transform the program. Despite encountering challenges related to effectiveness, evidence requirements, and integration within the healthcare system, the DiGA program continues to evolve and serves as a seminal example for the integration of DTx, offering valuable insights for healthcare systems globally.

## The German DiGA program as a nationwide example of a regulated Digital Therapeutics reimbursement pathway

Digital Therapeutics (DTx), usually regulated as Software as a Medical Device^1^, are evidence-based digital products which by treating a ”disease, disorder, condition, or injury” aim to have “demonstrable positive therapeutic impacts on patient health”^2^. While the concept of leveraging digital solutions for extending the reach of medical practitioners dates back to at least the 1990s^3^, healthcare systems worldwide continue to explore effective integration methods for these innovations^4,5^.

A notable instance of such integration is the 2019 Digital Healthcare Act in Germany^6,7^, which established a “Fast-Track” pathway^8^ for the evaluation and listing of selected DTx in a directory overseen by the German Federal Agency for Drugs and Medical Devices (BfArM)^9^. The Digital Health Applications, known in German as ‘Digitale Gesundheitsanwendungen’ (DiGA), once listed in this directory, automatically become eligible for prescription and reimbursement, benefiting over 74 million individuals covered by Germany’s public health insurances, called statutory sickness funds^10^. At the time of its implementation, this legislation was recognized internationally as the first pathway to reimburse DTx on a large scale^11^. Existing manuscripts have examined a variety of facets of the DiGA program, including evidence criteria and reimbursement^12–16^, physicians experiences^17,18^, and initial experiences across medical and informatic specializations^19–21^. This perspective aims to provide a summary of program’s development over three years, distilling key learnings and outlining the impending legislative changes that continue to shape this pathway for regulated digital health products.

To be eligible for inclusion in the BfArM DiGA directory, DTx products undergo a structured assessment that covers multiple criteria, including both technical and evidence-oriented ones^8^. The technical criteria mandate functionalities such as security, data protection as well as interoperability and currently require the classification as a lower-risk medical device, either class I or IIa, according to the European Medical Device Regulation (MDR) or Medical Devices Directive (MDD). Beyond these technical prerequisites, DTx applications must conduct comparative studies to demonstrate tangible positive care effects, which are defined in two ways: as a medical benefit, which refers to direct health improvements for patients, or as structural and procedural improvements, indicating patient-relevant enhancements in healthcare delivery.

**Fig. 1:**
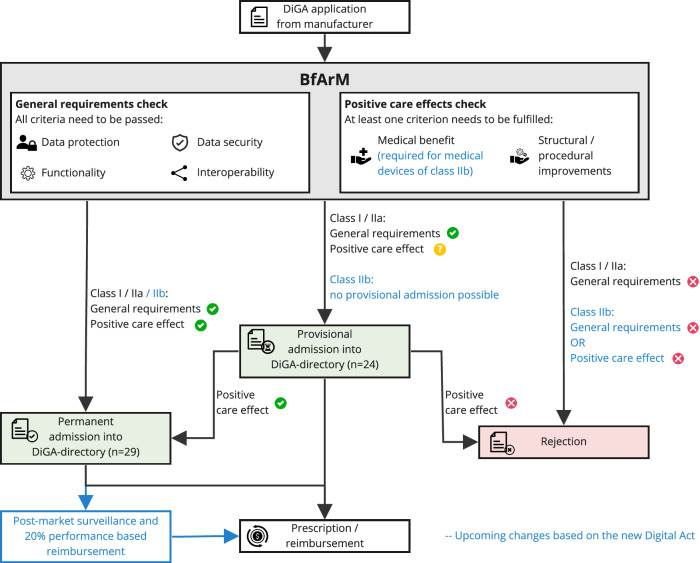
Current DiGA pathway and upcoming regulatory changes. Flowchart of the Digital Health Application (DiGA) “Fast-Track“ approval process, illustrating the steps from criteria checks to admission into the DiGA directory. Different pathways depending on the current evidence for the product and the class of the medical device are shown, with upcoming changes based on the new Digital Act depicted in blue.

Furthermore, the current process permits manufacturers to apply for a ‘provisional’ listing of their DiGA in the directory using preliminary evidence. Once provisionally listed, manufacturers have a twelve-month period, conditionally extendable, to produce high-quality evidence. During this time, the DiGA can be prescribed and is subject to conditional reimbursement (see Fig. 1). For the first year after listing, manufacturers have the flexibility to set their own prices within maximum limits determined by predefined rules, which consider the prices of comparable DiGAs. Under the agreement between insurers and manufacturers^22^, comparable DiGAs are categorized by their indication group and intended benefit, facilitating the establishment of maximum price reimbursement thresholds for each category that includes at least two DiGAs. In the first year of a DiGA’s listing, these maximum reimbursement limits are utilized as reference points for pricing, contingent upon the DiGA’s prescription volume. For DiGAs that pioneer their indication group, target rare diseases, or leverage sophisticated artificial intelligence, the standard pricing rules are modified, further incentivizing innovation and the addressing of unmet medical needs. Concurrently, negotiations between the manufacturer and the public insurances known as sickness funds take place to establish the price for the period following the initial 12 months, with these discussions also considering the established maximum reimbursement price. In cases where a price agreement is not reached, arbitration is used, with any determined prices applied retroactively from the 13th month onwards.

The pathway of preliminary DiGA listing is comparable to the already existing pathway of ‘new examination and treatment methods’, in German ‘Neue Untersuchungs- und Behandlungsmethoden’ (NUB), pathway, which allows for reimbursement of new examination and treatment methods that are not jet covered by the German Diagnosis Related Groups system for reimbursement. However, the provisional DiGA listing is strategically oriented to bridge the gap between innovation and evidence, while the NUB system aims to bridge the gap between the introduction of new examination and treatment methods and their formal integration into the reimbursement system.

## Three-year trends in DiGA applications since initiation of the program

**Fig. 2:**
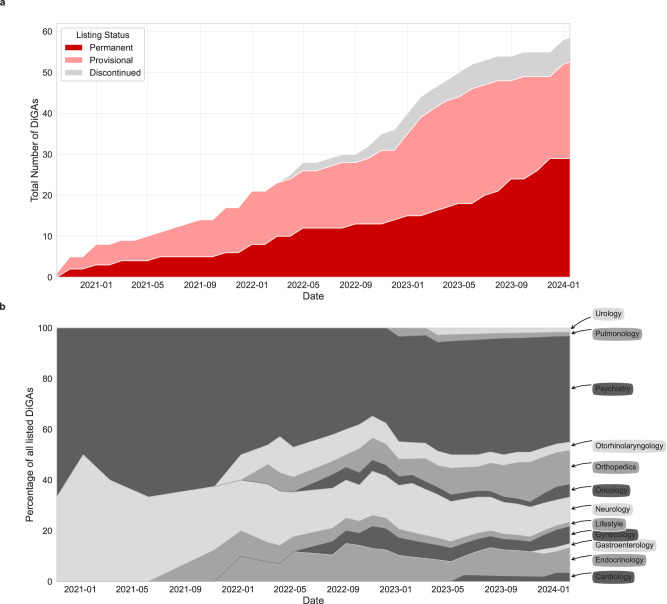
Number of DiGAs in different stages of the regulatory process and indication fields over time. **a** Stacked area chart showing the total number of Digital Health Applications (DiGAs) listed over time, categorized by listing status. The categories include provisional, permanent, and discontinued listings. **b** Stacked area chart showing the percentage of all DiGAs for all indication group over time, while each DiGA can count into multiple indication groups. All discontinued DiGAs are not included. Both graphs depict the listings from the directory’s initiation in 2020 to January 16^th^, 2024.

As of January 16th, 2024, the BfArM maintained directory listed 53 DiGAs, comprising 29 permanent listings and 24 provisional listings. Since its start in September 2020, six provisionally listed DiGAs have been removed from the directory, either due to failure in providing the required evidence or at the manufacturer’s request (see Fig. 2). The majority of DiGAs are available as mobile applications (34), often paired with a web application (9), while standalone web applications are less common (19). Over the three years since the program’s inception until the end of September 2023, approximately 374,000 DiGA prescriptions have been activated for patient use, reflecting an increasing trend and costing the German public health insurances approximately 113 million €^23^.

**Fig. 3:**
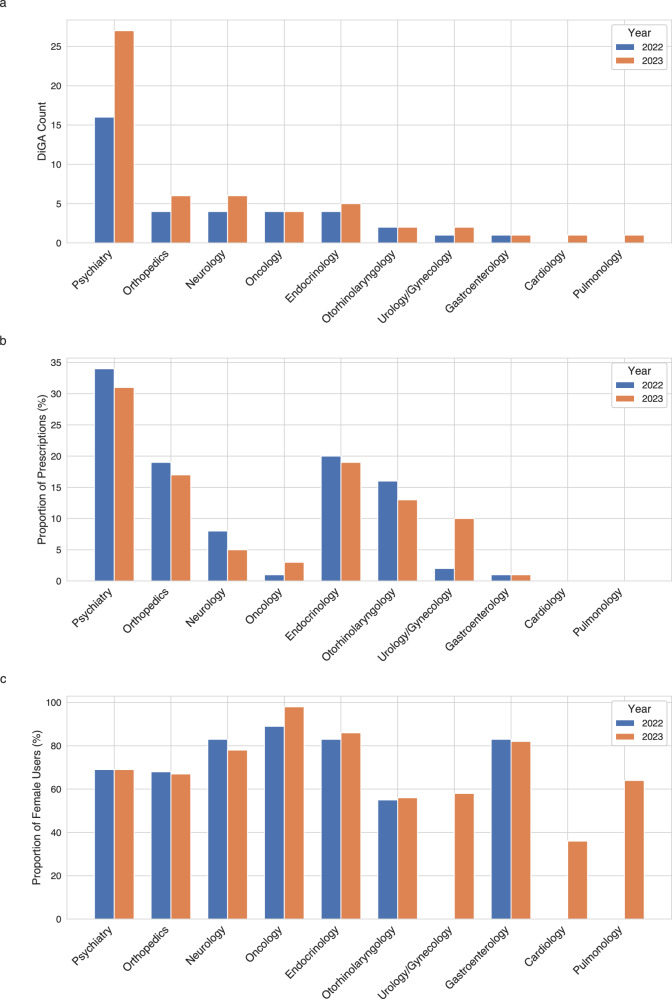
DiGA prescriptions and proportion of female users by indication group and year (2022 and 2023). **a** Digital Health Application (DiGA) count in 2022 and 2023 divided by indication group. **b** Proportion of prescriptions from the overall prescription in the respective years (2022 and 2023) by indication group. **c** Proportion of female users for each indication group in 2022 and 2023. All three subplots are based on the data from the official DiGA reports by the sickness fund association (GKV Spitzenverband)^23,41,42^. The data cut-off date is September 20^th^ of the respective years.

The directory predominantly features DTx for mental health conditions (25), followed by musculoskeletal disorders (8), neurological diseases (6), and endocrinological conditions (6), reflecting broader trends in DTx research^24^. Less common, yet represented, indication fields currently featured are gynecological diseases (4), oncology (3), diseases of the auditory system (2), cardiovascular diseases (2), respiratory diseases (1), urological diseases (1), lifestyle conditions (1), and diseases of the gastrointestinal tract (1) (7 DiGAs are listed in two separate indication groups). Initially, the directory focused mainly on psychiatric and neurological disorders, but over time, the range of covered indications has shown a diversifying trend (see Fig. 2). Interestingly, in terms of yearly prescription percentages, psychiatry leads, followed by endocrinology, orthopedics and diseases of the auditory system (see Fig. 3).

Most DiGAs offer a combination of lifestyle or condition-specific advice, cognitive behavioral therapy elements, informational features, tracking capabilities, and physiotherapy programs. Only a few utilize external sensors although the addition of external sensors to a DiGA has been permitted since the introduction of the DiGA pathway^25^. A notable trend is that most DiGA users are female, particularly in oncology, neurology, endocrinology, and gastroenterology applications. This reflects the broader pattern of women more frequently taking part in DTx trials^26^ and more often engaging in medical care services^27^. Recent findings in the US reveal that women engage more with health digitalization, seen in their greater use of wearables and openness to health data sharing^28^. While this may explain the higher DiGA usage among women, understanding the underlying reasons and addressing the imbalance requires further research.

In 2023, the median age across all indication groups spanned from 41 to 66 years^23^. Furthermore, most applications are class I medical devices, with only two classified as class IIa. Notably, five manufacturers list multiple DiGAs, with the highest number being seven from one manufacturer (GAIA AG), typically within similar indication areas (see Suppl. Figure 1).

**Fig. 4:**
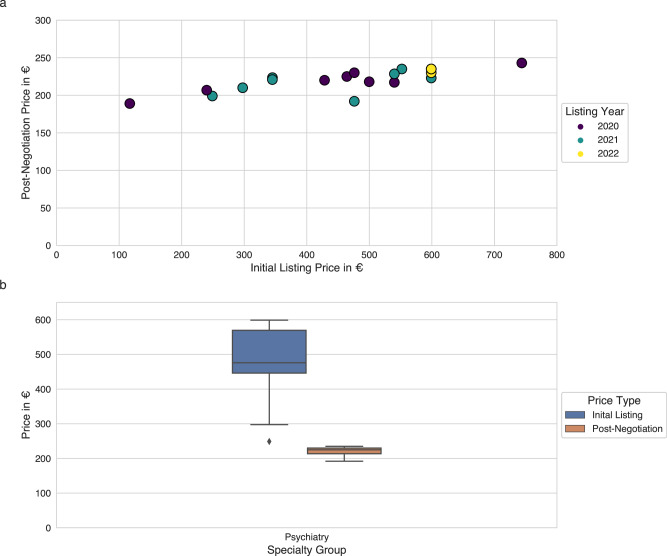
Initial manufacturer-set and final post-negotiation prices of permanently listed DiGAs. **a** Scatter plot illustrating the relationship between initial listing price and post-negotiation prices of 20 permanently listed Digital Health Applications (DiGAs) after price negotiation and with a 90-day prescription window across different listing years (at January 16th, 2024). Each point represents an individual DiGA, with the color indicating the year of initial listing. The x-axis denotes the initial listing price in euros set by the manufacturers, while the y-axis shows the negotiated permanent listing price in euros. DiGA prescribed via a one-time license are excluded. **b** Boxplots showing the variability of prices before and after price negotiation for the indication group psychiatry (*n* = 11) for DiGAs which completed the entire process. DiGAs prescribed via a one-time license are excluded. All other indication groups had a count of less than 4 DiGAs. DiGA manufacturers are within certain rules allowed to set a price for the first 12 months the app is listed in the directory, from the 13th month on a price negotiated between sickness funds and manufacturer applies. The boxplot center line indicates the median, box limits indicate upper and lower quartiles, whiskers indicate 1.5x interquartile range. The black point indicates an outlier.

The majority of permanently listed DiGAs are prescribed for a period of 90 days (28 of 29, with the notable exception of “Mawendo”, offering a one-time license), which is likely preferred due to its alignment with the quarterly billing cycle in German medical practices. After the initial 90 days of prescription, four DiGAs are offered for a discounted subsequent prescription. For permanently listed DiGAs with a 90-day prescription window and concluded negotiations with the sickness funds (*n* = 20) the mean cost for manufacturer-set prices for initial prescription is 465.42€, while for negotiated final prices it is 220.79€ (see Fig. 4). Although DiGA prices have been reported to steadily increase since their initial implementation^23^, there has been a shift towards more stable pricing, with a noticeable plateau beginning in 2023 at median of 540.00€ for manufacturer-set prices (see Suppl. Figure 2). This might be attributed to legislative changes in 2022 that imposed constraints on manufacturer-set prices. It should be highlighted that there exists a significant positive correlation between the initial prices set by manufacturers and the prices established post-negotiation (Pearson correlation coefficient of 0.79, *P* value < 0.001). Nonetheless, the absolute difference in negotiated prices, ranging from the highest at €243.00 to the lowest at €189.00, is relatively modest. Only one DiGA (“Kalmeda”) negotiated a higher permanent listing price than initially set by the manufacturers. Although, it is worth mentioning that the negotiated price is still below the overall average. The remaining 19 DiGAs negotiated a lower permanent price. While there is currently only one permanently listed DiGA with a one-time license, this appears to be a more common pricing model in more recently provisionally added DiGAs (5 of 24). The analysis of the additional devices (such as sensors) used in DiGAs, along with their corresponding medical device classifications and application platforms (web-based, mobile, or both) reveals no evident trend in the years following the program’s initiation.

## Criticisms of the DiGA program and current legislative changes aiming to reshape it

Throughout its three-year existence, the DiGA directory has seen both successes and controversies, exemplified by the ongoing discussions between public insurance companies and manufacturers. The reimbursing German sickness funds have expressed concerns over what they perceive as low effectiveness and insufficient evidence for DiGAs^23^, especially those provisionally listed in the directory, a sentiment echoed in some scientific literature^29^. On the other hand, DiGA manufacturers have highlighted challenges related to delayed patient access to applications, with a survey of 17 manufacturers indicating an average waiting time of 13 days post-prescription submission to the health insurance^30^. Manufacturers claim that this delay compromises the quick DiGA access for patients that is vital for treatment efficacy and acceptance.

Additionally, while alternative pricing models based on patient application usage or real-world effects are explicitly allowed in the regulation^8^, none of the DTx currently listed in the DiGA directory utilizes models such as treatment success-based reimbursement. Only the application “Smoke Free” offers a 7-day prescription-free start phase of all app functionalities. In general, the potential of DiGAs in collecting real-world data (RWD) and real-world evidence (RWE) for health technology assessment or flexible pricing remains largely untapped as of today^13,15^. This is despite the fact that DTx such as DiGAs, as digital tools, are uniquely equipped to gather patient-reported outcome measures (PROMs) and usage or performance data with minimal additional effort.

Other points of critique include the fact that initial analyses reveal mixed app-store reviews^31^ and emerging data in specific areas like rheumatology indicate limited adherence to DTx and varying user satisfaction^19^. Furthermore, a comprehensive analysis conducted by a leading German sickness fund, which combined routine data with a survey of over 1700 patients who used a DiGA, paints an ambivalent picture. While satisfaction with DiGAs among patients was high, they noted limited impact on the disease course and treatment outcomes, underlining the need to track efficacy for these DTx. A common concern was the lack of adequate information about the DTx, especially from prescribing physicians^17^.

The recent passage of two new laws by the German Bundestag, the Digital Act^32^ and the Health Data Use Act^33^, is set to significantly advance digitalization in the German healthcare system. Both laws will also bring fundamental changes to the DiGA program. Notably, future DiGAs can also include higher-risk medical devices, namely those classified as class IIb of MDR. This expansion, addressing previous criticisms of the program’s limited initial approach^11^, paves the way for more complex telemonitoring or remote monitoring approaches to be integrated into DTx using the DiGA pathway. However, in line with these changes, the legislator is imposing stricter evidence requirements for these higher-risk devices, such as the need for prospective comparative studies. Importantly, the option of ‘provisional listing’ for class IIb devices, based solely on preliminary evidence, will no longer be available^32^.

Another key forthcoming change in the DiGA program is the alteration of reimbursement methods, as from 2026 on at least 20% of a DiGA’s reimbursement price will be contingent upon success measures. While these specific measures are still to be defined by the BfArM, likely candidates may include adherence rates, user satisfaction, or other PROMs. Depending on the measures that will be assessed, this change could lead to an unprecedented mandatory generation of DiGA-specific RWD^13^ via PROMS and similar instruments, offering insights into actual usage and effectiveness of these DTx. This development is poised to contribute significantly to the ongoing discourse on value-based pricing, focusing on the balance of cost and effectiveness^16^. As RWD collection will subsequently be a feature embedded into all DiGAs, the further usage of RWE^13^ for assessment of DiGAs appears also more likely.

Additionally, an initial proposal for a 14-day test phase, allowing patients to try a DiGA and opt-out without incurring reimbursement costs, was considered in the early drafts of the new legislation but ultimately not adopted in the final version^32,34^. Such concepts generally raise the question, whether DTx should be treated differently than other therapeutics such as pharmaceutical or surgical interventions, where test phases would be impossible to realize.

Additional significant changes have been incorporated into the final legislation with the aim to streamline DiGA access and integration within Germany’s digital health ecosystem. One such change mandates that sickness funds must provide access to DiGAs within a maximum of two days. Moreover, there is an emphasis on enhancing DiGA integration into the broader digital health infrastructure, which includes a new requirement for interoperability with the electronic health record system starting later in 2024. This system, despite its current infrequent usage, is set to become more widely adopted as it will be rolled out as an opt-out solution for all publicly insured individuals in Germany by the beginning of 2025^32,34^.

Although more than half of general practitioners in Germany have already prescribed DiGAs^17^, recent research has further highlighted the critical importance of digital health literacy and the affinity of practicing doctors towards digital technologies for the success of digital health adoption^35^. This research further points to the essential need for enhancing public awareness and developing comprehensive digital education programs for physicians^36^. As the DiGA-manufacturing companies rapidly expand their workforce^30^, it is likely that currently often lacking sales and distribution networks will be established and consequently further contribute to public and healthcare provider awareness.

While recent evolutions in the DiGA program are designed to address many existing criticisms (see Table 1 for key learnings and legislative changes), certain issues remain unaddressed. The dynamic nature of DiGAs (and generally all DTx), as updatable software products, presents challenges to the current methods of evidence generation and health technology assessment, which may not be ideally suited for such evolving products^14^. Furthermore, the lack of international cooperation and standardized frameworks not only limits the transferability of innovations across borders but consequently also leads to economic inefficiencies^37^. While there is still much to be done in this area, initial promising efforts to harmonize standards such as the European Taskforce for Harmonised Evaluations of Digital Medical Devices^38,39^, led by the French Ministry of Health and Prevention and coordinated by EIT Health, should be supported and encouraged to ensure the international transferability of DTx and other digital tools.

**Table 1:** Selected key learnings and future legislative changes addressing these

Key Learning	Legislative Change Addressing the Learning
Restricting approvals to only medical devices of class I or IIa narrows the range of potential DiGA applications.	Future regulations will permit class IIb medical devices to qualify as DiGA, albeit with stricter requirements, such as the necessity for demonstrated medical benefits and no accelerated track.
There appears to be a discrepancy between DiGA pricing and their clinical benefits, indicating that the actual success of a DiGA is not adequately reflected in its cost.	Starting in 2026, a minimum of 20% of a DiGA’s reimbursement price will be linked to the achievement of (yet to be defined) success metrics.
Patient access to prescribed DiGA is often delayed, highlighting inefficiencies in delivery systems.	Health insurances will be mandated to provide access to DiGA within two days of prescription, aiming to reduce patient waiting times.
DiGA integration into the broader German healthcare system is currently insufficient.	Increased requirements for DiGA integration into electronic health records systems are anticipated, with an emphasis on interoperability.
A significant number of users discontinue using their prescribed DiGA shortly after initial registration or first use, suggesting issues with user engagement or application efficacy.	A proposed 14-day trial period for DiGA was included in the first versions of the new legislation, but was not included in the final draft.

Meanwhile, the DiGA program, with its ongoing revisions and updates, serves as a notable example of how DTx can be successfully integrated into a healthcare system that has historically been slow to adopt digitalization^40^. Indeed, the DiGA program’s core principles are being replicated in other healthcare settings, most notably by France, which in 2023 introduced a comparable “Fast-Track” initiative for DTx and telemedicine applications, notably across all MDR risk classes^4^. Belgium, with a different reimbursement system, also introduced a ‘Fast-Track’ for DTx, which builds on a pyramidal assessment of technical and evidence prerequisites^4^.

In summary, the use of DTx, as illustrated by the DiGA case, faces multi-level challenges. Physicians need to integrate DTx, PROMs, and monitoring into their routines effectively, while more research is required to enhance patient adherence to DTx and to understand the observed usage disparities across genders. The collaboration between payers and manufacturers is essential for DTx success, particularly as this nascent market and emerging field continues to develop its processes and priorities.

The recent adjustments to the German DiGA framework signal a promising shift aiming at greater impact for patients and underline the adaptive process necessary when introducing piloting legislature. By incorporating higher-risk devices and mandating the collection of RWD, the German DiGA program is poised for significant evolution. It thus continues to stand as a leading model for implementing DTx at scale, providing globally valuable insights into the challenges, opportunities, and potential pitfalls encountered along the way.

### Supplementary information

Supplementary Material

## Data Availability

The data underlying this work are from public sources, explicitly the DiGA directory^9^, the DiGA reports by the sickness fund association^23,41,42^ and public data aggregated in the “DiGA Analyzer” by _fbeta GmbH^43^.

## Code availability

Final data and code used for designing the graphs can be accessed in the following GitHub repository: https://github.com/Linea-code/DiGA_analysis_figures.
