# Supplementary material for: The three-year evolution of Germany’s Digital Therapeutics reimbursement program and its path forward
Source: NPJ Digit Med. 2024 May 24;7:139. doi: 10.1038/s41746-024-01137-1 (PMC11126413; doi:10.1038/s41746-024-01137-1)
Supplement: Supplementary file 1 — Supplementary Material [file 41746_2024_1137_MOESM1_ESM.pdf]

Supplementary Figures

Supplementary Figure 1

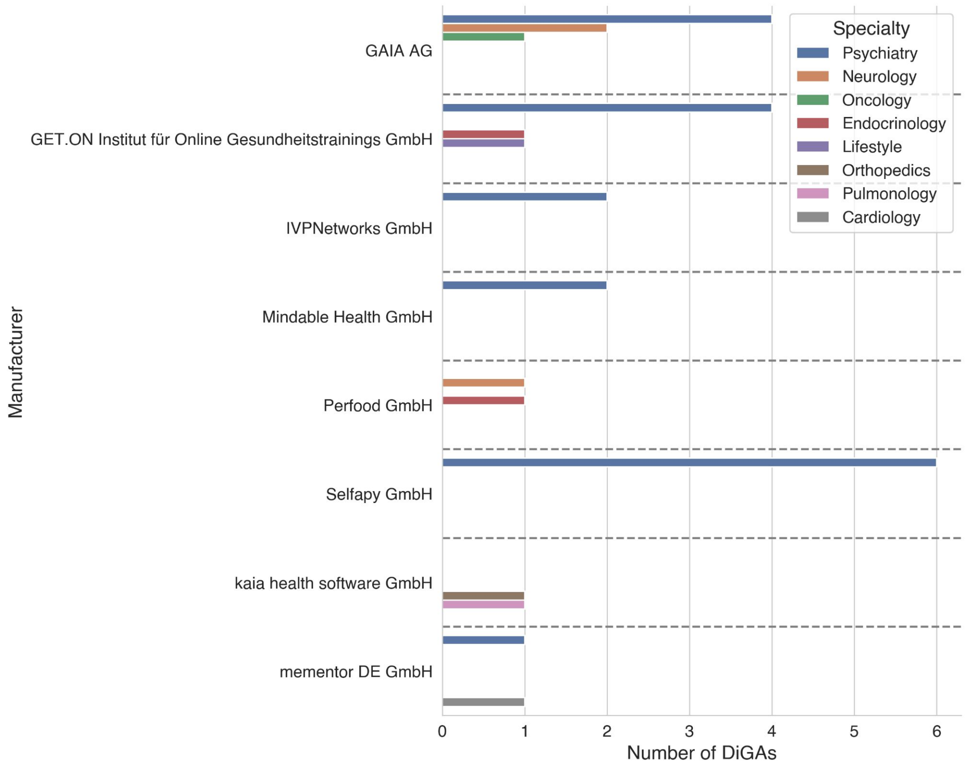

Suppl. Figure 1: Bar plot depicting the overall count of DiGAs per specialty and manufacturer. Discontinued DiGAs are counted as well. DiGAs for more than one specialty (n=5) are counted in only one specialty group.

Supplementary Figure 2

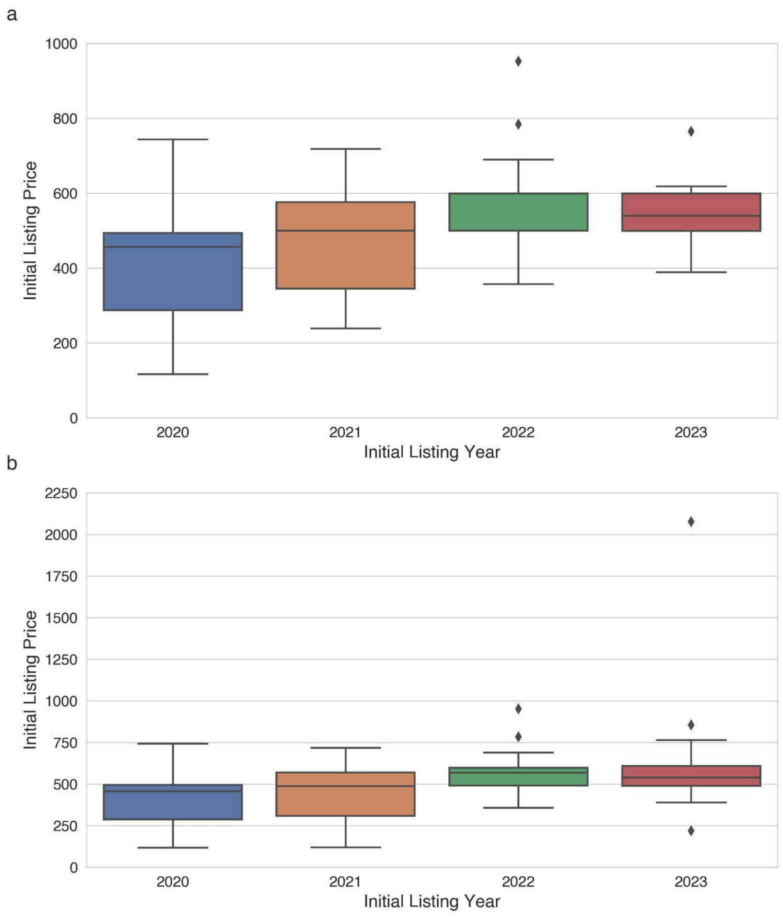

Suppl. Figure 2: Boxplots representing the price distributions of initial listing prices of DiGAs per year from 2020 until 2023 by depicting the median, interquartile range and whiskers (1.5x the interquartile range). a) The plot is based on all DiGAs with a 90-day prescription window and non-one-time license applications, independent of their current or past listing status. b) The plot is based on all DiGAs independent of their current or past listing status or prescription time and licensing model.
